# Genome sequences of 19 *Klebsiella* phages of the genus *Przondovirus*

**DOI:** 10.1128/mra.00211-26

**Published:** 2026-05-18

**Authors:** Tracey L. Peters, Caitlin D. Urick, Amanda M. Ward, Kevin A. Burke, Jamie L. Braverman, Matthew T. Koert, Olga A. Kirillina, Nino Mzhavia, Mikeljon P. Nikolich, Andrey A. Filippov

**Affiliations:** 1 Department of Animal, Veterinary and Food Sciences, College of Agricultural and Life Sciences, University of Idaho 5640, Moscow, Idaho, USA; 2 Wound Infections Department, Bacterial Diseases Branch, Walter Reed Army Institute of Research 8394, Silver Spring, Maryland, USA; Katholieke Universiteit Leuven, Leuven, Belgium

**Keywords:** *Klebsiella pneumoniae*, *Przondovirus*, phage, genomes

## Abstract

Here, we describe the genomes of 19 *Klebsiella* phages of the genus *Przondovirus*. The ranges of genome lengths, GC content, and numbers of predicted protein-coding genes were 39,014–41,853 bp, 52.4–53.5%, and 53–61, respectively. Based on key parameters, these phages appear to be lytic and suitable for therapeutic use.

## ANNOUNCEMENT

Phages are used to successfully treat multidrug-resistant (MDR) *Klebsiella pneumoniae* infections (1). We are developing therapeutic phage cocktails against MDR bacterial pathogens, and here report the genomes of 19 phages isolated on diverse *K. pneumoniae* strains, mainly MDR clinical isolates (Table 1).

**TABLE 1:** Genomic properties of 19 *K. pneumoniae* phages of the genus *Przondovirus*

Phage full name	Short name	Isolation date	Host strain	Raw read no.	Average read coverage	Genome length, bp	GC (%)	No. of CDSs^*a*^	BACPHLIP score	BioSample accession no.	SRA accession no.	GenBank accession no.
vB_Kpn9997-EKp2	EKp2	9/14/16	ATCC 9997	2,171,118	7,937	39,371	53.3	56	99.73	SAMN53084708	SRR35954259	PX694429
vB_Kpn419800-EKp24	EKp24	10/31/18	MRSN 419800	311,730	1,815	41,484	53.3	56	97.31	SAMN53084709	SRR35954260	PX694421
vB_Kpn533757-EKp38	EKp38	11/21/19	MRSN 533757	532,250	2,199	40,491	52.9	59	100.00	SAMN53084710	SRR35954261	PX694426
vB_Kpn546043-EKp39	EKp39	11/21/19	MRSN 546043	54,996	316	40,428	53.0	59	99.87	SAMN53084711	SRR35954262	PX694427
vB_Kpn18776-EKp44	EKp44	11/21/19	MRSN 18776	193,322	1,306	41,853	52.9	57	100.00	SAMN53084712	SRR35954263	PX694419
vB_Kpn27617-EKp55	EKp55	11/21/19	MRSN 27617	216,326	1,484	40,927	53.1	59	99.87	SAMN53084713	SRR35954264	PX694420
vB_Kpn430405-EKp91	EKp91	11/21/19	MRSN 430405	442,396	3,458	40,656	53.0	58	99.81	SAMN53084714	SRR35954265	PX694422
vB_Kpn508238-EKp110	EKp110	11/21/19	MRSN 508238	420,580	2,583	40,273	53.1	61	98.48	SAMN53084705	SRR35954273	PX694423
vB_Kpn533757-EKp119	EKp119	11/21/19	MRSN 533757	415,416	2,303	40,428	53.3	55	99.73	SAMN53084706	SRR35954257	PX694425
vB_Kpn582610-EKp146	EKp146	04/07/22	MRSN 582610	587,030	3,829	40,894	53.0	54	99.87	SAMN53084707	SRR35954258	PX694428
vB_Kpn101683-EKp159	EKp159	3/6/24	MRSN 101683	965,970	5,114	39,814	53.0	54	100.00	SAMN53084696	SRR35954275	PX694411
vB_Kpn101683-EKp160	EKp160	3/6/24	MRSN 101683	702,386	5,330	40,116	52.4	57	99.87	SAMN53084697	SRR35954274	PX694412
vB_Kpn102803-EKp162	EKp162	3/6/24	MRSN 102803	898,266	6,863	39,014	53.1	53	99.73	SAMN53084698	SRR35954266	PX694413
vB_Kpn107743-EKp166	EKp166	6/27/23	MRSN 107743	1,201,380	8,962	41,015	53.0	53	100.00	SAMN53084703	SRR35954271	PX694418
vB_Kpn106667-EKp167	EKp167	6/27/23	MRSN 106667	1,226,302	8,494	40,438	52.8	55	99.73	SAMN53084699	SRR35954267	PX694414
vB_Kpn106667-EKp168	EKp168	6/27/23	MRSN 106667	1,198,022	5,934	40,590	53.5	56	100.00	SAMN53084700	SRR35954268	PX694415
vB_Kpn107119-EKp172	EKp172	3/6/24	MRSN 107119	89,938	638	40,848	53.0	56	100.00	SAMN53084701	SRR35954269	PX694416
vB_Kpn107119-EKp173	EKp173	3/6/24	MRSN 107119	119,488	908	40,722	53.0	54	100.00	SAMN53084702	SRR35954270	PX694417
vB_Kpn517281-EKp181	EKp181	3/6/24	MRSN 517281	839,494	6,056	40,546	53.0	60	92.50	SAMN53084704	SRR35954272	PX694424

^*a*^ CDSs, protein-coding sequences.

Phages were isolated from sewage collected either in Washington, DC (GPS coordinates: 38.819362, 77.022137, phages EKp2 through EKp119) or in Montgomery County, MD, USA (GPS coordinates: 39.142441, −77.276509, all the other phages). Phage enrichment was performed in broth, then phage plaques were picked from double-layer agar plates and phages purified by three single-plaque isolations (2). Phage DNA was extracted from lysates with the QIAamp DNA Mini Kit (Qiagen, Germantown, MD, USA). Libraries were prepared using the KAPA HyperPlus Kit (Roche Diagnostics, Indianapolis, IN, USA) and sequenced on an Illumina MiSeq (Illumina, San Diego, CA, USA) with a 600-cycle MiSeq Reagent Kit v3 that produced 300 bp paired-end reads.

The quality of paired-end reads (Table 1) was evaluated and trimmed using Fastp (3) v1.0.1. Where needed, reads were subsampled using seqtk (https://github.com/lh3/seqtk) v1.4 (accessed on 2 October 2025) to achieve ~100× of expected genome length. Trimmed reads were assembled using Unicycler (4) v0.5.0, and genome termini were assessed by PhageTerm (5) v3.0.1. Phage lifestyle was predicted with BACPHLIP (6) v0.9.6. Protein and tRNA coding sequences (CDSs) were annotated using the Pharokka pipeline (7–18) v1.6.0. Phage contigs were matched to their closest hits in the INPHARED database (17) using mash (18) v2.2.2. Default parameters were used for all programs.

**Fig 1:**
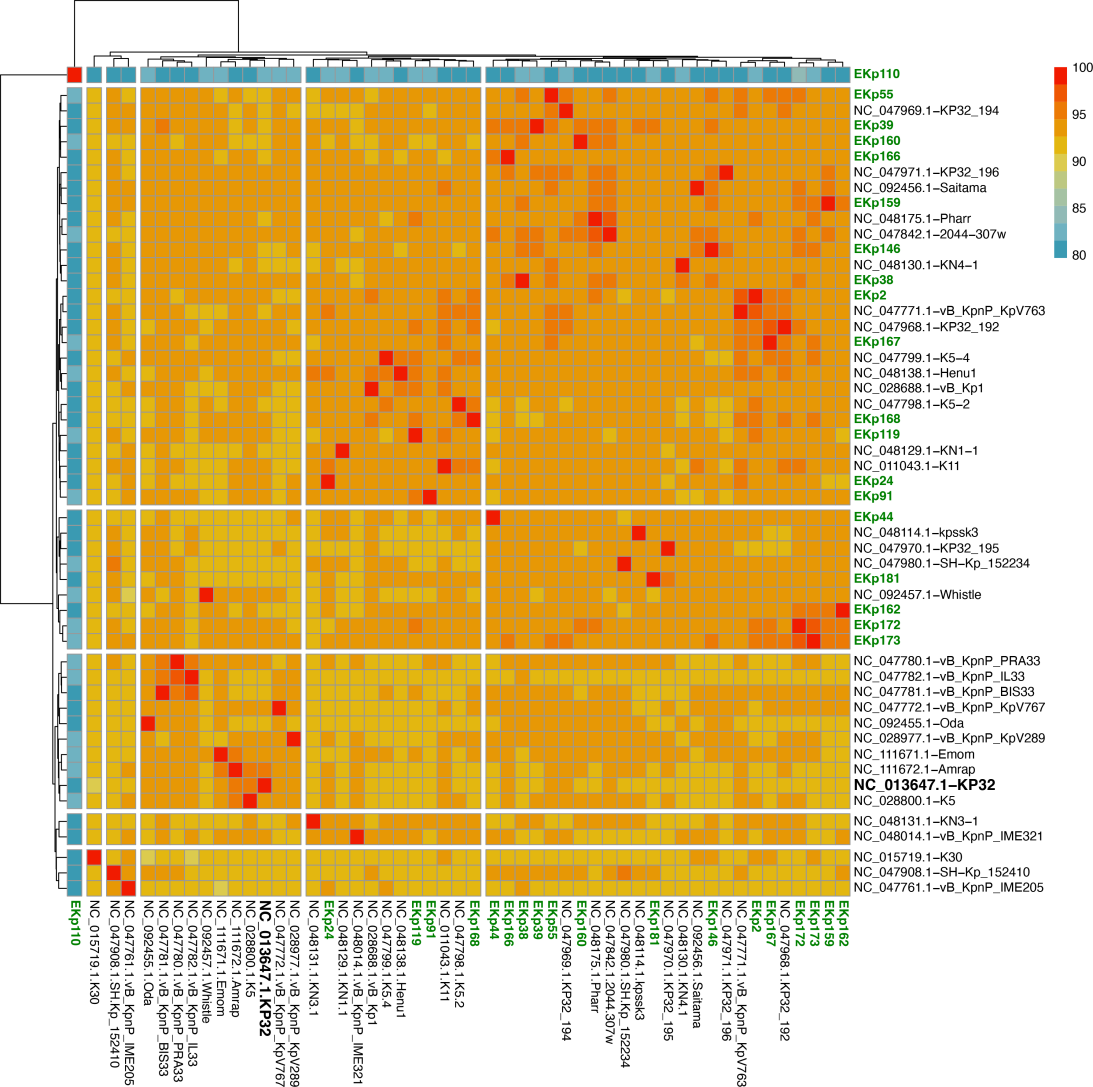
Heatmap representing wgANI of 19 *Przondovirus* phages (marked in bold green) and 32 NCBI RefSeq *Przondovirus* genomes listed by ICTV, with *Przondovirus* phage KP32 (NC_013647.1) as the type species (marked in bold black). The figure is based on wgANI calculations from fastANI and generated using pheatmap v1.0.13 in R.

Average read coverages for the 19 *Klebsiella* phage genomes varied from 316× to 8,962×. The genome lengths, GC contents, and numbers of CDSs were in the ranges 39,014–41,853 bp, 52.4–53.5%, and 53–61, respectively (Table 1). PhageTerm (5) identified direct terminal repeats of 133–215 bp. No tRNA genes were found in these genomes. Mash alignment (18) against the INPHARED database (17) placed these phages in the family *Autotranscriptaviridae*, subfamily *Studiervirinae*, genus *Przondovirus*. Pairwise comparisons using FastANI v1.34 revealed 80.03–97.12% whole-genome average nucleotide identity (wgANI) (19) to 32 RefSeq *Przondovirus* genomes that are listed in the ICTV Master Species List (https://ictv.global/msl, MSL40.v2, accessed 3 November 2025), including the type species *Przondovirus* KP32 (NC_013647.1) (Fig. 1). Podophages of this prevalent group have demonstrated environmental stability (20), relatively broad host ranges (21), anti-biofilm activity (20), synergy with antibiotics (21, 22), and efficacy against *K. pneumoniae* infection in waxworm (20) and mouse lung (22), sepsis (21), and intestinal colonization (23) models.

BACPHLIP (6) scored these 19 phages at 93–100%, suggesting a lytic lifestyle. No homology was found between their putative proteins and products involved in lysogenicity, gene transfer, or bacterial proteins, such as drug resistance determinants (10) and virulence factors (11). Thus, these 19 *Przondovirus* phages appear to be lytic and promising therapeutic candidates.

## Data Availability

Data for the phage genomes are available through NCBI, under BioProject accession number PRJNA1357368; BioSample, GenBank, and the NCBI Sequence Read Archive accession numbers are listed in Table 1.
